# Molecular mechanisms of genetic variation and transcriptional regulation of *CYP2C19*

**DOI:** 10.3389/fgene.2012.00206

**Published:** 2012-10-10

**Authors:** Nuala Ann Helsby, Kathryn Elisa Burns

**Affiliations:** Molecular Medicine and Pathology, Faculty of Medical and Health Sciences, University of AucklandAuckland, New Zealand

**Keywords:** *CYP2C19*, pharmacogenetics, transcription factors, epigenetics, miRNA

## Abstract

Inherited variation in the function of the drug metabolizing enzyme CYP2C19 was first observed 40 years ago. The SNP variants which underpin loss of CYP2C19 function have been elucidated and extensively studied in healthy populations. However, there has been relatively meagre translation of this information into the clinic. The presence of genotype-phenotype discordance in certain patients suggests that changes in the regulation of this gene, as well as loss of function SNPs, could play a role in deficient activity of this enzyme. Knowledge of the molecular mechanisms which control transcription of this gene, reviewed in this article, may aid the challenge of delivering *CYP2C19* pharmacogenetics into clinical use.

## Introduction

CYP2C19 is important in the metabolism of many clinically relevant drugs (Desta et al., 2002), particularly for several prodrugs that require hepatic activation including clopidogrel (Bauer et al., 2011; Begg et al., 2012). The first reports of an autosomal recessive inherited trait that resulted in poor metabolism of the prototypical CYP2C19 substrate, mephenytoin, appeared many years ago (Kupfer et al., 1979). The correlation between the poor metabolizer phenotype and loss of function genotype has been comprehensively studied in healthy populations (Desta et al., 2002). Screening for these genetic variants is one approach to individualize therapy for drugs which are substrates for this enzyme. However to be useful genotype must be predictive of phenotype, not only in healthy populations but also in the clinical context (Helsby, 2008). The presence of genotype-phenotype discordance in certain morbidities (Williams et al., 2000; Frye et al., 2002; Helsby et al., 2008), suggests that an acquired deficiency in the activity of this enzyme also occurs. Factors which regulate *CYP2C19* transcription could play an additional role in the pharmacogenomic variation of this enzyme. The current knowledge of the molecular mechanisms which control expression of the *CYP2C19* gene, such as the coding and regulatory region *cis*-variants as well as *trans*-acting epigenomic factors, are reviewed in this article.

## *CYP2C19* gene variants

The role of *cis*-acting variants of *CYP2C19* have been extensively characterized and the polymorphic expression of these genetic variants results in inter-individual variation in CYP2C19 activity. More than 28 variant alleles in *CYP2C19* have been identified (http://www.cypalleles.ki.se/, access date August 19, 2012) and are summarized in Table 1. Many of these variants have relatively low frequency however, the SNPs which lead to the CYP2C19^*^2 and CYP2C19^*^3 alleles are common and have been the most extensively studied. These SNPs, c.681G>A, and c.636G>A, cause aberrant splicing and a premature stop codon respectively and result in null function. Individuals who are homozygous variant for either of these alleles are poor metabolizers of certain drugs. The allele frequency of these null function variants varies with ethnicity (Xie et al., 2001; Sistonen et al., 2009). A particularly high prevalence of both ^*^2 and ^*^3 is observed in Vanuatu and Papua New Guinea, accounting for up to 70.8% (^*^2) and 13.3% (^*^3) of these alleles in the Vanuatu population, and up to 42.3% and 31.5%, respectively, in the Papua New Guinea population (Kaneko et al., 1997; Hsu et al., 2008). This exceptionally high expression in Melanesia may reflect an unidentified evolutionary pressure. For further information on these null function alleles and the drug substrates of CYP2C19 readers are directed to review articles that have focused on *CYP2C19* pharmacogenetics such as Desta et al. (2002). In addition to genetic variation in the coding region of the gene, promoter region variation may also influence transcriptional expression and ultimately activity. The CYP2C19^*^17 allele (g.–3402C>T and g.–806C>T) has been the focus of studies to identify increased function variants of this gene. Ethnic variation in the prevalence of this allele is also observed with a relatively low allele frequency (<5%) in Japanese and Chinese populations, compared with a higher incidence in European and African populations (~15–30%) (Li-Wan-Po et al., 2010).

**Table 1:** ***CYP2C19* gene variants**.

**Allele**	**Characteristic SNP^a^**			**Functional change**	**References**
	**cDNA**	**Gene**	**Effect**		
CYP2C19^*^1	None*^1^*	None	None	Normal	Romkes et al., 1991
CYP2C19^*^2	681G>A*^2^*	19154G>A	Splicing defect	Non-functional	De Morais et al., 1994b; Ibeanu et al., 1998b; Fukushima-Uesaka et al., 2005; Lee et al., 2009; Satyanarayana et al., 2009a
CYP2C19^*^3	636G>A*^3^*	17948G>A	Premature stop codon (W212X)	Non-functional	De Morais et al., 1994a; Fukushima-Uesaka et al., 2005
CYP2C19^*^4	1A>G*^4^*	1A>G	GTG initiation codon	Non-functional	Ferguson et al., 1998; Scott et al., 2011
CYP2C19^*^5	1297C>T*^5^*	90033C>T	R433W	Non-functional	Xiao et al., 1997; Ibeanu et al., 1998a
CYP2C19^*^6	395G>A	12748G>A	R132Q	Non-functional	Ibeanu et al., 1998b
CYP2C19^*^7		19294T>A	Splicing defect	Non-functional	Ibeanu et al., 1999
CYP2C19^*^8	358T>C	12711T>C	W120R	Decreased *in vitro*	Ibeanu et al., 1999
CYP2C19^*^9	431G>A	12784G>A	R144H	Decreased *in vitro*	Blaisdell et al., 2002
CYP2C19^*^10	680C>T	19153C>T	P227L	Decreased *in vitro*	Blaisdell et al., 2002
CYP2C19^*^11	449G>A	12802G>A	R150H	Similar to wild type *in vitro*	Blaisdell et al., 2002
CYP2C19^*^12	1473A>C	90209A>C	X491C; 26 extra amino acids	Unstable *in vitro*	Blaisdell et al., 2002
CYP2C19^*^13	1228C>T	87290C>T	R410C	Similar to wild type *in vitro*	Blaisdell et al., 2002
CYP2C19^*^14	50T>C	50T>C	L17P	Not determined	Blaisdell et al., 2002
CYP2C19^*^15	55A>C	55A>C	I19L	Not determined	Blaisdell et al., 2002
CYP2C19^*^16	1324C>T*^6^*	90060C>T	R442C	Not determined	Morita et al., 2004
CYP2C19^*^17		3402C>T; −806C>T		Increased transcription *in vitro*; Should not be termed Ultrarapid (UM)	Sim et al., 2006
CYP2C19^*^18	986G>A	80156G>A; 87106T>C	R329H	Not determined	Fukushima-Uesaka et al., 2005
CYP2C19^*^19	151A>G	151A>G; 87106T>C	S51G	Not determined	Fukushima-Uesaka et al., 2005
CYP2C19^*^20^7^	636G>A	17948G>A	Premature stop codon (W212X) and D360N	Non-functional	Fukushima-Uesaka et al., 2005
CYP2C19^*^21^8^	681G>A	19154G>A; –98T>C	splicing defect and A161P	Non-functional	Fukushima-Uesaka et al., 2005; Satyanarayana et al., 2009a
CYP2C19^*^22	557G>C	17869G>C	R186P	Not determined	Matimba et al., 2009
CYP2C19^*^23	271G>C	12455G>C	G91R	Not determined	Zhou et al., 2009
CYP2C19^*^24	1004G>A; 1197A>G	80174G>A; 87259A>G	R335Q	Not determined	Zhou et al., 2009
CYP2C19^*^25	1344C>G	90080C>G	F448L	Not determined	Zhou et al., 2009
CYP2C19^*^26	766G>A	19239G>A	D256N	Decreased *in vitro*	Lee et al., 2009
CYP2C19^*^27		–1041G>A		Decreased *in vitro*	Drögemöller et al., 2010
CYP2C19^*^28	1120G>A	−2020C>A; −1439T>C; 80290G>A	V374I	No significant decrease *in vitro*	Drögemöller et al., 2010

a Only major SNP or alteration(s) responsible for the phenotype of the corresponding allele are shown. Adapted from http://www.cypalleles.ki.se/

1 The presence of additional SNP can further sub-classify individuals as ^*^1B (99C>T; 991A>G) or ^*^1C (991A>G). This results in an I331V change but does not alter activity.

2 The presence of additional SNP can further sub-classify individuals as ^*^2A, ^*^2B, ^*^2C, and ^*^2D. Of these variants ^*^2C and ^*^2D harbor a SNP in the 5′ promoter region (−98T>C) that may have a functional effect.

3 The presence of additional SNP can further sub-classify individuals as ^*^3A (1251A>C) and ^*^3B (1078G>A; 1251A>C).

4 The presence of −3402C>T; −806C>T SNP in the promoter can further sub-classify individuals as ^*^4B.

5 The presence of 99C>T; 991A>G, can further sub-classify individuals as ^*^5B.

6 Existence of the CYP2C19 ^*^2 polymorphism 681G>A on the same allele cannot be excluded.

7 Also known as CYP2C19 ^*^3B.

8 Also known as CYP2C19 ^*^2C.

## Regulatory polymorphisms

### CYP2C19^*^17

CYP2C19^*^17 was first identified in 2006 (Sim et al., 2006). Electrophoretic mobility shift assays (EMSA) detected binding of human hepatic nuclear proteins at −806T but not −806C. A potential GATA binding site at this position was identified *in silico* and it was hypothesized that the −806C>T variant could result in increased transcription of *CYP2C19.* However, to date GATA-dependent transactivation at the −806C>T site has not been directly demonstrated. Indeed it is of note that following co-transfection with GATA-4 or GATA-6, −806T>C variant reporter constructs did not have increased luciferase activity compared with wildtype constructs (Mwinyi et al., 2010b). Although GATA may not be involved, transfection of reporter constructs of the −0.9 Kb of the 5′ flanking region into mice lead to an increase in transcription in the −806T mutant compared with wildtype construct. However a range of overlapping individual luciferase activities were observed in wildtype and mutant constructs. To date no direct evidence of correlations between CYP2C19^*^17 genotype status and increased transcription or protein expression in human liver biobanks has been reported. Despite the lack of direct evidence this genotype is often described as increasing the expression of the enzyme (protein) and many investigators have categorized individuals who carry this variant allele as ultra-rapid metabolizers (UM). When CYP2C19 activity is measured *in vivo* using drug to metabolite ratios of probe substrates such as omeprazole it is clear that the mean activity of CYP2C19 is higher in homozygous ^*^17/^*^17 subjects than in individuals with the ^*^1/^*^1 genotype (Baldwin et al., 2008). However, the activity in ^*^17/^*^17 subjects overlaps the heterogenous activity observed in ^*^1/^*^1 subjects (Baldwin et al., 2008). Similar effects have been observed with other drugs such as escitalopram, clopidogrel and voriconazole (Li-Wan-Po et al., 2010). The high activity in some ^*^1/^*^1 subjects may be due to other currently unidentified increased activity variants. However currently ^*^17/^*^17 subjects do not appear to be a separate population and fall within the normal distribution of wildtype CYP2C19 activity, therefore should not be classified as an ultra-rapid phenotype. Moreover, the ultra-rapid metabolizer phenotype observed with CYP2D6 substrates is typically due to gene duplication and associated copy number variation, and it is important to note that copy number variation for *CYP2C19*, appears to be absent (Drögemöller et al., 2010; Devendran et al., 2012). Further identification of additional SNP in the 5′-up-stream region of *CYP2C19* may clarify the wide heterogeneity of activity in ^*^1/^*^1 individuals.

## Other promoter region variants

Publication of the promoter sequence of *CYP2C19* gene (Genbank accession #AF354181) led to the identification of eight SNP in the −1.833 Kb promoter region (Arefayene et al., 2003). Resequencing of genomic DNA from 92 individuals of varied ethnicity identified 13 SNP in the −1.46 Kb up-stream region of the gene (Blaisdell et al., 2002). Extensive characterization of the 5′-regulatory region of *CYP2C19* also identified a further seven SNP novel variants in the enhancer region and five SNP in the promoter region in Japanese subjects (Fukushima-Uesaka et al., 2005). Eight novel SNP were also detected in the −1.7 Kb promoter region in a South Indian population (Satyanarayana et al., 2009b). More recently resequencing of −2.095 Kb of the 5′-up-stream region of *CYP2C19* identified two additional novel SNP (g.−2030C>T and g.−2020C>A). These SNPs, in combination with a previously identified SNP in the 5′promoter (g.−1439T>C) and the g.80290 G>A SNP in exon 7, result in the CYP2C19^*^28 genotype (Table 1) (Drögemöller et al., 2010). Extensive ethnic variation in the frequency of promoter/enhancer region SNP is evident from the above studies. Identification of the functional effects of these 35 novel SNP identified up-stream of the translational start site is important for our understanding of the variable expression and activity of this enzyme.

Regions of negative and positive regulatory control of *CYP2C19* were observed following transient expression of luciferase reporter deletion constructs in HepG2 cells (Arefayene et al., 2003). Transient expression into HepG2 cells of luciferase reporter deletion constructs between positions −153 bp and −17 bp significantly decreased luciferase activity, suggesting effects on transcription factor binding. In contrast deletion from −650 bp to −363 bp increased luciferase activity, indicating the presence of repressor regulation in this region. Using nine different constructs of the *CYP2C19* 5′ promoter region (−1.6 Kb) transfected into HepG2 cells, Satyanarayana et al. (2011) showed that the presence of either the −98T>C SNP in combination with −1498T>G or the combination of −98T>C, −779A>C, −1051T>C, and −1418C>T, significantly increased luciferase activity. The SNP −98T>C is within both a potential CCAAT displacement protein (CDP) binding site and a potential GATA-1 site (Satyanarayana et al., 2009b). *In silico* analysis indicated that interaction of the CDP repressor with its putative binding site was weaker in the presence of the −98C variant whereas GATA-1 had a high predicted binding activity in the presence of the normal −98T. Hence the functional consequence may be that in wildtype subjects (−98TT or CT) repression of GATA-1 binding will be greater than in homozygous −98CC subjects. Indeed, the presence of −98TT genotype appears to decrease the activity of the enzyme compared with the −98CT or −98CC genotype in subjects probed *in vivo* with proguanil (Satyanarayana et al., 2009a). This is suggestive of a functional effect of a transcriptional repressor at this region of the promoter, in agreement with the early data from Arefayene et al. (2003). It is of interest to note that −98T>C displays linkage with the c.681G>A (^*^2) SNP (Fukushima-Uesaka et al., 2005; Satyanarayana et al., 2009b) as CYP2C19^*^21, also known as CYP2C19^*^2C (Table 1).

Most recently the g.−1041G>A SNP in the CYP2C19^*^27 allele (Table 1) has also been demonstrated to have functional consequences. Significantly decreased luciferase activity was observed in a construct transfected into HepG2 cells, whereas the promoter region SNP (−2030C>T;−1439T>C) in the CYP2C19^*^28 allele did not significantly decrease activity (Drögemöller et al., 2010). Thus, to date only three of the 35 SNP identified in the proximal five region of *CYP2C19* appear to be associated with changes in gene transcription: −806C>T (^*^17), −1041G>A (^*^27) and −98T>C (^*^21).

## Transcription factor binding sites in *CYP2C19*

**Figure 1:**
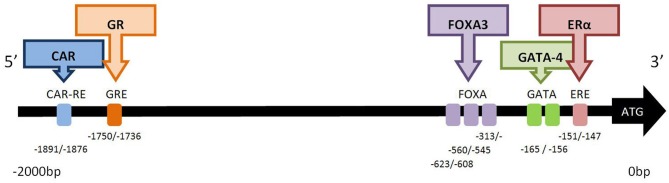
**Functional transcription factor binding sites in the 2.0 kb 5′-promoter region of *CYP2C19.*** The binding factor CAR (constitutive androstane receptor; NR113) acts at the CAR response element (−1891/−1876 bp). NR113 can also hetero-dimerise with retinoid X receptor (RXR) and pregnane X receptor (PXR) to transactivate *CYP2C19* (Chen et al., 2003). The glucocorticoid receptor (GR; NR3C1) acts at the glucocorticoid response element (GRE) at position −1750/−1736 bp (Chen et al., 2003). Hepatocyte nuclear factor 3 gamma (HNF3γ) is member of the forkhead box/winged helix family of transcription factors and is also known as FOXA3. Three functional FOXA3 sites, (−313/−298, −560/−545, −623/−608 bp) exist in the *CYP2C19* promoter (Bort et al., 2004). GATA-4 is a member of the zinc-finger transcription factor family. Two adjacent GATA binding motifs (TATC) are found between −165/−156 bp, and GATA-4 predominantly binds to site I (−165/−162 bp). The repressor protein, Friend of GATA (FOG-2), attenuates the effect of GATA-4 binding (Mwinyi et al., 2010b). Estrogen receptor-α (ERα) binds to the estrogen receptor element (ERE) half site at −151/−147 bp. Mutation of this site decreases but does not abolish transcription (Mwinyi et al., 2010a).

Many predicted or putative sites for transcription factor binding have been reported for *CYP2C19*. However, functional transcription factor binding sites have only been demonstrated for the ligand activated nuclear receptors ERα (NR3A1) (Mwinyi et al., 2010a), CAR (NR1I3), and GR (NR3C1) (Chen et al., 2003), and the transcription factors HNF3γ (FOXA3) (Bort et al., 2004) and GATA-4 (Mwinyi et al., 2010b), (Figure 1).

The constitutive androstane receptor response element (CAR-RE) at −1891/−1876 in the promoter region of *CYP2C19* has been shown to functionally active. Binding of CAR protein occurs as a monomer or heterodimer with the retinoid X receptor (RXR) and pregnane X receptor (PXR). Deletion of this site completely abolishes binding (Chen et al., 2003). Expression of *CYP2C19* appears to be more sensitive to the effects of co-transfection with CAR than with PXR, nevertheless luciferase activity can be induced by the PXR activator rifampicin (Chen et al., 2003), a known inducer of CYP2C19 activity in patients (Feng et al., 1998). There is a significant correlation between *CAR* mRNA and *CYP2C19* transcription in human liver (Wortham et al., 2007). Deletion constructs have demonstrated the functional activity of a glucocorticoid responsive element (GRE) at −1750/−1736b, and dexamethasone can also induce the expression of a *CYP2C19* construct containing this GRE in transfected HepG2 and Caco2 cells (Chen et al., 2003).

A functional estrogen response element (ERE) half site has been identified in *CYP2C19* at position −151/−147 (Mwinyi et al., 2010a). This GGTCA motif binds ERα but not ERβ. Both 17-β estradiol and 17-α ethinylestradiol down-regulate reporter luciferase activity in Huh-7 transfected cells co-transfected with ERα. However, the partial agonists 4-hydroxytamoxifen and raloxifene had no effect on *CYP2C19* transcription (Mwinyi et al., 2010a). Interestingly mutation of this ERE half site decreases but does not abolish luciferase activity. Chromatin immunoprecipitation of Huh-7 cells combined with q-PCR demonstrated that ERα was associated with this ERE half site in the *CYP2C19* promoter. 17-α ethinylestradiol stimulated this interaction of ERα with the promoter whereas 4-hydroxytamoxifen abolished the interaction. Preliminary data suggested that treatment of primary hepatocytes with 17-β estradiol or 17-α ethnylestradiol decreased *CYP2C19* mRNA expression (Mwinyi et al., 2010a). This is in contrast to recent data which found that estradiol did not influence the expression of *CYP2C19* (Choi et al., 2012). The biotransformation of estrogens by CYP enzymes results in a half-life of estradiol in human hepatocytes of <34 min. Thus concentration dependent ligand binding may influence transactivation of *CYP2C19* by ERα in hepatocytes.

In addition to the three ligand-activated transcription factors described above, two additional transcription factor proteins have also been demonstrated to be important in *CYP2C19* transcription:- FOXA3 and GATA-4. Three hepatocyte nuclear factor 3 gamma (HNF3γ) sites, (−313/−298, −560/−545, −623/−608), have been identified in the *CYP2C19* promoter (Bort et al., 2004). Cotransfection with HNF3γ in luciferase reporter assays as well as overexpression of HNF3γ in HeLa and hepatoma cells significantly increased expression of *CYP2C19* (Bort et al., 2004). HNF3γ is member of the forkhead box/winged helix family of transcription factors and is also known as FOXA3. This transcription factor is important in the expression of liver-specific genes and the development of hepatic lineage. In contrast, co-transfection with HNF4α, did not increase luciferase expression despite the presence of HNF4α sites at −186/−174, −152/−140 (Kawashima et al., 2006). These sites do not appear to be functional as deletion constructs, indicating that HNF4α cannot increase transcription of *CYP2C19* (Bort et al., 2004) and also indicate that HNF4α protein does not bind to *CYP2C19* (Bort et al., 2004; Rana et al., 2010).

GATA-4 is a member of the zinc-finger transcription factor family. Two adjacent GATA binding motifs (TATC) have been detected in the *CYP2C19* promoter at −165/−162 and −159/−156 (Mwinyi et al., 2010b). Wildtype and deletion constructs containing destructive mutations in each of the GATA sites were transfected into HepG2 or Huh-7 cells. Significant up-regulation of luciferase activity of these constructs was observed when co-transfected with either GATA-4 or GATA-2. Deletion of this double GATA binding site completely abolished transcription. However, EMSA analysis demonstrated nuclear extracts predominantly bind to site I (−165/−162) and chromatin immunoprecipitation confirmed that GATA-4 was associated with the *CYP2C19* promoter. The GATA repressor protein friend of GATA 2 (FOG-2), also known as zinc finger protein multitype-2, attenuates the effect of GATA-4 and may also have a role to play in the regulation of *CYP2C19* transcription. GATA-4 is an important liver associated transcription factor (Molkentin, 2000).

More recently, it has been proposed that the following transcription factors and binding sites may also be important in the transcription of *CYP2C19*:- ATF-2 (−806 to −786), CEBP-β (−1505/−1491 and −1443/−1429), CDP repressor protein (−105/−87), GATA-1 (−103/−91) and an additional GRE (−828/−810), however further functional analysis is required to establish the relevance, if any, of these factors (Satyanarayana et al., 2011). The weak correlations between *PXR, ARNT*, and *HNF1α* genes with *CYP2C19* mRNA expression in human liver suggests that these transcriptional regulators do not extensively contribute to *CYP2C19* expression (Wang et al., 2011).

## *Trans* acting factors

In contrast to *cis* regulation due to genetic variants, the *trans*-acting factors which control *CYP2C19* expression have been largely ignored. Knowledge of epigenomic control of *CYP2C19*, via factors such as altered expression of transcription factor genes or the effects of noncoding RNA, is limited. A number of studies have identified that environmental (rather than inherited genetic) effects such as pregnancy, old age, cancer, and congestive heart failure (Williams et al., 2000; Frye et al., 2002; McGready et al., 2003; Ishizawa et al., 2005) can all lead to an acquired alteration in CYP2C19 activity. The observed change in activity can lead to genotype-phenotype discordance, such that a poor metabolizer status can be observed in individuals who are not homozygous variant for null function alleles. This may be due to *CYP2C19* gene down-regulation as has been observed following incubation of the inflammatory cytokines, IL-6 and TGF-β, with primary human hepatocytes (Aitken and Morgan, 2007). The mechanisms for this down-regulation of *CYP2C19* have not been elucidated to date but could include direct effects on gene transcription (e.g., CpG methylation), up-stream effects on the expression or activity of transcription factors or post-translational regulation of *CYP2C19*.

## Epigenetics

Tissue specific regulation of genes can be the result of epigenetic regulation and it is notable that the expression of *CYP2C19* is restricted to the liver and intestine (Läpple et al., 2003; Hayashi et al., 2011; Bourgine et al., 2012). Quantification of gene transcripts (cDNA copy number) indicates the same range and median expression of *CYP2C19* mRNA in the intestine as in the liver. However, the authors could not detect an intra-individual correlation between *CYP2C19* expression in samples of liver and intestine from each patient expression. Hence there may be independent regulation of transcription of *CYP2C19* in these tissues (Läpple et al., 2003). One possible mechanism of tissue specific expression is epigenetic control via methylation of CpG islands in the gene or by histone modifications such as acetylation.

**Figure 2:**
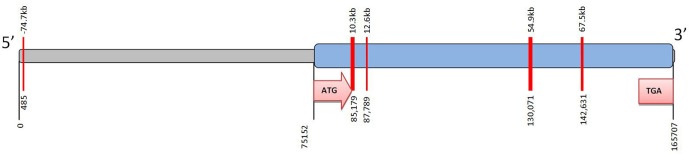
**CpG islands identified in *CYP2C19*.** The complete *CYP2C19* sequence (Ensembl Gene ID ENSG00000165841; National Centre for Biotechnology Information (NCBI); Entrez core nucleotide sequence NM 000769) was analyzed using CpG Island finder, http://cpgislands.usc.edu/. Potential CpG island regions were determined using the following parameters %GC = 50–55%, ObsCpG/ExpCpG = 0.6, length = 100–500 bp, gap between adjacent islands = 100 bp. Up to five CpG islands were identified in *CYP2C19*. Notably most of these CpG islands are down-stream of the ATG initiation codon (i.e., within the coding region of the gene) and are not associated with the 5′proximal promoter. The CpG islands are shown as red bars at 485, 85179, 87789, 130071, and 142631 bp. The 5′ upstream region is shown in grey and the coding region of the gene in blue.

Remarkably little is known about epigenetic regulation of *CYP2C19* (Ingelman-Sundberg et al., 2007). However a small number of CpG islands can be detected in the gene (Figure 2). Methylation of CpG sites in the promoter region of a target gene can affect the physical binding of transcription factors to regulate gene expression. In the case of *CYP2C19* these CpG islands are not associated with the promoter region. However, DNA methylation can also act via an indirect mechanism on chromatin configuration. There is currently no data regarding the methylation status of the *CYP2C19* CpG islands.

DNA methylation can lead to a phenomenon known as allelic expression imbalance. The difference in expression levels between two alleles of *CYP2C19* typically occurs due to genetic polymorphisms (e.g., a heterozygous carrier of ^*^3). However epigenetic silencing of an allele (e.g., methylation) can also result in the preferential expression of one of the two alleles. Allelic expression imbalance is assessed directly by the relative quantitation of an intragenic marker allele in cells or tissues. In the absence of any *cis*-acting control on transcription the allelic ratio should be 1 (i.e., equal amounts from each allele). In liver samples from heterozygous individuals, the *cis*-acting variant of *CYP2C19* (^*^2; rs4244285) accounts for the majority of the allelic expression imbalance observed. However, there was only a weak correlation (*p* = 0.047) between *CYP2C19* mRNA expression and this SNP in these 96 human livers. This suggests that variability in *CYP2C19* expression is not fully accounted for by known coding region polymorphisms. Interestingly, the non-coding marker SNP in intron 3, (rs 4388808), was associated with up to 47% of allelic expression imbalance for *CYP2C19* (Wang et al., 2011). This confirms that in addition to *cis*-acting polymorphic variants, there are factors which influence the regulatory control of *CYP2C19* RNA transcription or stability. Hence epigenetic factors may also affect the hepatic expression level of *CYP2C19*.

## Post-translational regulation of *CYP2C19*

In addition to transcriptional regulation, post-transcriptional regulation may influence the expression of *CYP2C19*. Noncoding RNA, such as microRNA (miR), can bind to recognition sites (MRE) in the 3′-untranslated region (3′UTR) or in the coding region of target genes and thereby repress gene translation. The role of miR regulation of *CYP2C19* was until recently not known, as *in silico* prediction of miR regulation of *CYP2C19* was not available due to the lack of information about the 3′-UTR of the gene (Ramamoorthy and Skaar, 2011). However, it has recently been reported that the 3′UTR of *CYP2C19* contains two putative MRE for miR-103/107 at 222–242 bp and 138–152 bp down-stream of the stop codon. These MRE contain one nucleotide mismatch, however, ectopic addition of precursors of miR-103/107 to human hepatocytes significantly down-regulated CYP2C19 immunoreactive protein (Zhang et al., 2012). This preliminary data suggests that post-transcriptional regulation of the constitutive expression of *CYP2C19* may be an additional contributing factor to inter-individual variation in the expression of this enzyme in subjects who do not express SNP variants.

## Control of transcription factor activity

Another mechanism which could account for variation in the regulation of *CYP2C19* expression is the effect of both genomic and environmental factors which influence transcription factor-binding to the promoter region. SNP variants present in the promoter region have been discussed above, however up-stream effects on the expression or activity of transcription factors may also play a role in *CYP2C19* transcription. It is important to appreciate that as well as altered expression of transcription factor genes the function of ligand-activated factors (ERα, CAR, GR) can be influenced by variation in the levels of endogenous ligands, such as estrogens and glucocorticoids. Hence environmental factors may influence the activity of transcription factors important for *CYP2C19* transcription. The ability of ERα, CAR, GR, FOXA3, and GATA-4 to interact with other transcription factors may add further complexity to the regulation of *CYP2C19*. For example, estrogen-dependent activation of ERα results in binding to the ERE. This appears to result in a down-regulation of *CYP2C19* transcription (Mwinyi et al., 2010a). Changes in CYP2C19 activity have been reported in women during pregnancy and whilst using oral contraceptives (McGready et al., 2003), suggesting a regulatory role for estrogens on CYP2C19 activity. However, ligand-independent activation of ERα also occurs. ERα can act via a non-classical pathway to alter the activities of other transcription factors (e.g., Sp1, AP-1, or NF-kappaB) at their cognate sites on DNA. The role of interactions of ER-α with other transcription factors that regulate *CYP2C19* cannot be discounted. This may account for why mutation constructs of the ERE site decrease but do not abolish *CYP2C19* transcription in the presence of ligand activated ER-α (Mwinyi et al., 2010a). In addition, GATA-4 co-operates with FOXA3 to stimulate albumin gene transcription in liver cells (Cirillo et al., 2002). Moreover, FOXA3 and GATA-4 can act as pioneer factors. Once bound these pioneer factors relax the adjacent chromatin to allow other factors to bind. Interestingly GATA-4 appears to be able to direct the association of ERα in certain contexts (Miranda-Carboni et al., 2011).

Understanding the genomic control of *CYP2C19* expression is important in order to increase our understanding of the observed phenotype-genotype discordance in morbidity. This acquired deficiency may influence the sensitivity and specificity of *CYP2C19* pharmacogenetic tests in clinical contexts. A correlation between *CYP2C19* mRNA and the expression of *CYP2C9* and *CYP3A4* has been observed (Wang et al., 2011) and Bayesian network analysis suggests that *CYP2C19* is the master regulator of *CYP2C9, CYP3A7, CYP3A4*, and *CYP3A43* (Yang et al., 2010). Hence further study of the mechanisms which regulate *CYP2C19* may also increase our understanding of the regulation of other important drug metabolizing enzymes.

### Conflict of interest statement

The authors declare that the research was conducted in the absence of any commercial or financial relationships that could be construed as a potential conflict of interest.
